# High Plasma Levels of Fibroblast Growth Factor 23 Are Associated with Increased Risk of COVID-19 in End-Stage Renal Disease Patients on Hemodialysis: Results of a Prospective Cohort

**DOI:** 10.3390/toxins15020097

**Published:** 2023-01-19

**Authors:** Luis Toro, Luis Michea, Alfredo Parra-Lucares, Gabriel Mendez-Valdes, Eduardo Villa, Ignacio Bravo, Catalina Pumarino, Patricia Ayala, María Eugenia Sanhueza, Ruben Torres, Leticia Elgueta, Sebastian Chavez, Veronica Rojas, Miriam Alvo

**Affiliations:** 1 Division of Nephrology, Department of Medicine, Hospital Clinico Universidad de Chile, Santiago 8380456, Chile; 2 Centro de Investigación Clínica Avanzada, Hospital Clínico Universidad de Chile, Santiago 8380456, Chile; 3 Fuerza de Trabajo Anti-COVID-19 (FUTAC Team), Sociedad Chilena de Nefrología, Santiago 7500781, Chile; 4 Programa de Fisiología y Biofísica, ICBM, Facultad de Medicina, Universidad de Chile, Santiago 8380453, Chile; 5 Division of Critical Care Medicine, Department of Medicine, Hospital Clinico Universidad de Chile, Santiago 8380456, Chile; 6 MD PhD Degree Program, Faculty of Medicine, Universidad de Chile, Santiago 8380456, Chile; 7 School of Medicine, Faculty of Medicine, Universidad de Chile, Santiago 8380456, Chile; 8 Division of Internal Medicine, Department of Medicine, Hospital Clinico Universidad de Chile, Santiago 8380456, Chile

**Keywords:** end-stage renal disease, hemodialysis, FGF23, COVID-19, SARS-CoV-2, immune response, mortality

## Abstract

End-stage renal disease (ESRD) patients are a population with high rates of COVID-19 and mortality. These patients present a low response to anti-SARS-CoV-2 immunization, which is associated with immune dysfunction. ESRD patients also present high plasma titers of Fibroblast Growth Factor 23 (FGF23), a protein hormone that reduces immune response in vivo and in vitro. Increased FGF23 levels associate with higher infection-related hospitalizations and adverse infectious outcomes. Thus, we evaluated whether ESRD patients with high FGF23 titers have an increased rate of SARS-CoV-2 infection. Methods: We performed a prospective cohort of ESRD patients in hemodialysis who had measurements of plasma intact FGF23 in 2019. We determined COVID-19 infections, hospitalizations, and mortality between January 2020 and December 2021. Results: We evaluated 243 patients. Age: 60.4 ± 10.8 years. Female: 120 (49.3%), diabetes: 110 (45.2%). During follow-up, 45 patients developed COVID-19 (18.5%), 35 patients were hospitalized, and 12 patients died (mortality rate: 26.6%). We found that patients with higher FGF23 levels (defined as equal or above median) had a higher rate of SARS-CoV-2 infection versus those with lower levels (18.8% versus 9.9%; Hazard ratio: 1.92 [1.03–3.56], *p* = 0.039). Multivariate analysis showed that increased plasma FGF23 was independently associated with SARS-CoV-2 infection and severe COVID-19. Discussion: Our results suggest that high plasma FGF23 levels are a risk factor for developing COVID-19 in ESRD patients. These data support the potential immunosuppressive effects of high circulating FGF23 as a factor implicated in the association with worse clinical outcomes. Further data are needed to confirm this hypothesis.

## 1. Introduction

The SARS-CoV-2 pandemic has had a massive impact worldwide over the past two years, with over 620,000,000 infections and over 6,500,000 deaths as of October 2022 [1]. Patients with end-stage renal disease (ESRD) in renal replacement therapy, such as renal transplantation, peritoneal dialysis, and hemodialysis, are a population with higher infection rates and adverse outcomes, including hospitalizations, requirements of mechanical ventilation and deaths, compared to the general population [2,3,4,5].

ESRD patients develop an immune dysfunction that includes impairment in both innate and adaptive immune response [6], including decreased neutrophil bacterial activity, monocyte hypoactivity, impaired activity of T lymphocytes, and a decreased number of B lymphocytes [6]. This immune impairment is associated with a higher rate of infections and up to 100-fold higher infection-related mortality than the general population [7]. ESRD patients also present a high failure rate of immunization against viruses such as influenza, despite appropriate vaccination [6], and immunization against SARS-CoV-2 produced a lower humoral response in ESRD patients versus control subjects [7]. Patients on hemodialysis who were vaccinated with mRNA vaccines induce anti-SARS-CoV-2 antibodies [8], although at lower concentrations than healthy volunteers [9]. In addition, they have an early decrease in antibody titers compared to the general population [10,11,12]. The causal mechanisms leading to the immune impairment of ESRD patients are not entirely understood.

ESRD patients present high plasma levels of Fibroblast Growth Factor 23 (FGF23), a protein hormone involved in the regulation of vitamin D and phosphate metabolism [13]. In addition to its role in mineral homeostasis, recent evidence shows that FGF23 acts as an immunomodulatory hormone, affecting the immune response due to indirect actions on non-immune tissues and direct actions on immune cells, including macrophages and polymorphonuclear neutrophils. Elevated titers of FGF23 are associated with increased inflammation in patients [14], and increased levels of FGF23 induce immune dysfunction in experimental murine models and human white blood cells in vitro [15]. In addition, clinical studies in ESRD patients [16] and non-ESRD chronic kidney disease patients [17] show that high plasma levels of FGF23 are associated with an increased infection rate compared to patients with lower titers.

We propose that high baseline plasma levels of FGF23 are associated with increased risk and severity of SARS-CoV-2 infection in the ESRD population. Therefore, we evaluated a multicenter prospective cohort of adult ESRD patients in chronic hemodialysis to determine the association between high FGF23 titers and the incidence of SARS-CoV-2 infection and COVID-related adverse clinical outcomes.

## 2. Results

### 2.1. Baseline Characteristics

We evaluated 243 end-stage renal disease patients on chronic hemodialysis for the study. Table 1 presents the baseline characteristics of these patients. Age: 60.4 ± 10.8 years. Patients over 60 years: 132 (54.3%). Female: 120 (49.3%). Diabetes: 110 (45.2%), hemodialysis vintage: 25 [15–40] months. Baseline intact FGF23 levels: 319 [204–600] pg/mL.

### 2.2. COVID-19-Related Events and Predictors of SARS-CoV-2 Infection

During follow-up (January 2020–December 2021), 45 patients (18.5%) had SARS-CoV-2 infection, and 35 patients (14.4%) presented COVID-related hospitalizations. Regarding mortality, 32 patients (13.1%) died during follow-up: 12 patients (4.9%) had COVID-related deaths, and 20 (8.2%) died of other causes, where cardiovascular diseases were the most frequent causes. Regarding the period of COVID-19 events, 40/45 of total SARS-CoV-2 infections (88.9%) and 10/12 of total COVID-related deaths (83.3%) occurred before the initiation of the vaccination campaign in hemodialysis patients (initiated in February 2021 in Chile).

When comparing patients who developed SARS-CoV-2 infection versus those without infection (Table 1), infected patients were older (66.2 ± 9.3 versus 59.1 ± 10.7 years, *p* < 0.001) and had a higher rate of diabetes (60.0 vs. 41.9%, *p* = 0.028). Concerning additional baseline variables before the SARS-CoV-2 infection, the only parameter which differed between both groups was plasma FGF23 which was higher in those who developed COVID-19 (436 [269–669] vs. 288 [195–580] pg/mL, *p* = 0.026). This association was sustained after statistical adjustment for age and other comorbidities.

### 2.3. Relation between Plasma FGF23 Levels and COVID-19-Related Events

**Figure 1:**
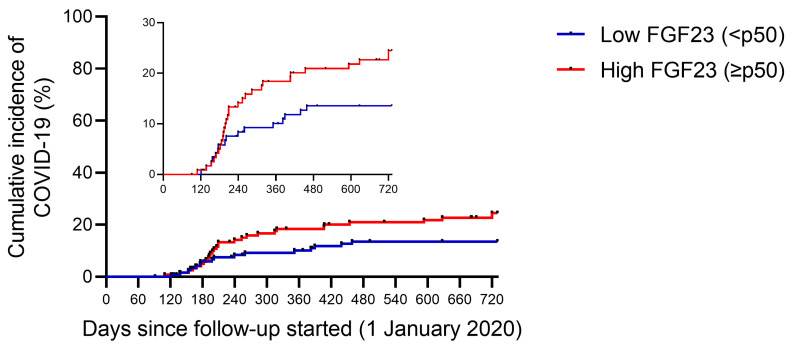
Infection rate of SARS-CoV-2 during follow-up, stratified by baseline plasma FGF23 levels. Cumulative incidence rate of SARS-CoV-2 infection in end-stage renal disease patients in hemodialysis. Patients were classified according to baseline plasma intact FGF23 levels: low FGF23 (n = 121—blue line) and high FGF23 (n = 122—red line).

**Figure 2:**
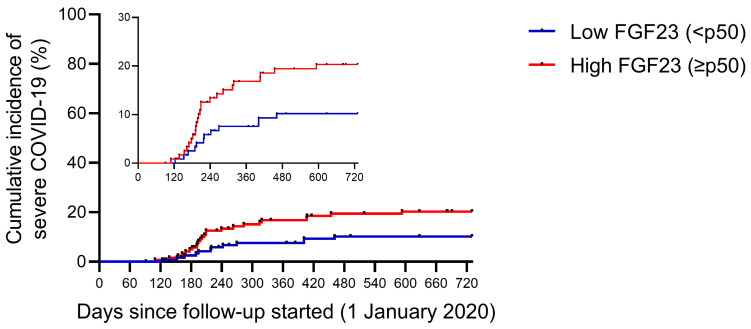
Severe COVID-19 rate during follow-up, according to baseline plasma FGF23 levels. Cumulative incidence of severe COVID-19 in end-stage renal disease patients in hemodialysis. Severe COVID-19 is defined as the requirements for hospitalizations or COVID-related deaths. Patients were classified according to baseline plasma intact FGF23 levels: low FGF23 (n = 121—blue line) and high FGF23 (n = 122—red line).

When comparing according to their baseline plasma FGF23 levels (Table 2), patients who had higher plasma FGF23 levels (equal to or above p50 of the total sample) had lower single-pool Kt/V (1.27 ± 0.20 versus 1.35 ± 0.21 years, *p* = 0.003), higher use of recombinant erythropoietin (88.5 vs. 78.5%, *p* = 0.035) and increased parathormone (PTH) levels (598 [465–703] vs. 204 [160–267] pg/mL, *p* < 0.001). In addition, people who had higher FGF23 titers had increased infection rates (23.7 vs. 13.2%, *p* = 0.034; Figure 1) and increased rates of severe COVID-19 (patients with COVID-related hospitalizations and deaths) (19.6 vs. 9.9%, *p* = 0.032; Figure 2).

Multivariate analysis indicates that SARS-CoV-2 infection predictors (Table 3) included age above 60 years (HR: 2.63 [1.35–5.11], *p* = 0.004), presence of diabetes (HR: 1.92 [1.05–3.48], *p* = 0.033) and high plasma FGF23 levels (HR: 1.92 [1.03–3.56], *p* = 0.039). No association was detected with another baseline clinical and laboratory variables. Finally, multivariate analysis indicates that the predictors of severe COVID-19 (Table 4) also included age above 60 years (HR: 2.65 [1.22–5.77], *p* = 0.014), presence of diabetes (HR: 2.52 [1.25–5.06], *p* = 0.009) and high plasma FGF23 levels (HR: 2.12 [1.04–4.28], *p* = 0.036).

## 3. Discussion

### 3.1. Association between Plasma Levels of FGF23 and COVID-19-Related Outcomes

This multicenter observational study showed that patients with end-stage renal disease in chronic hemodialysis who presented high baseline plasma levels of FGF23 had a higher rate of SARS-CoV-2 infection and severe COVID-19, including hospitalizations and deaths. In addition, this risk factor was independent of other variables associated with increased infections, such as age and diabetes. This study is one of the first clinical studies that evaluated the association between plasma FGF23 levels and COVID-19 in patients on renal replacement therapy. Currently, most studies evaluating the association between plasma FGF23 levels and infectious morbidity were performed before the SARS-CoV-2 pandemic and evaluated other infection-related hospitalizations caused by severe bacterial and viral infections [16,17]. A recent study determined that patients with asymptomatic SARS-CoV-2 infection had higher values of fibroblast growth factors (including FGF19, FGF21, and FGF23) compared to those with mild symptomatic COVID-19 [18].

### 3.2. Association between High Baseline FGF23 Levels and Development of Severe Infections in Humans

A posthoc analysis of the Hemodialysis Study (HEMO study) in ESRD patients on hemodialysis found that patients with high baseline FGF23 levels had an increased rate of a combined outcome of first infectious hospitalization or infectious death in a median follow-up of three years [16]. This association was independent of other associated variables. In addition, a posthoc analysis of the Chronic Kidney Insufficiency Cohort Study (CRIC study) in non-terminal chronic kidney disease (CKD) patients found that patients who had high baseline plasma titers of C-terminal FGF23 had an increased risk of first-time hospitalization with a severe infection, including pneumonia, urinary tract infection, and septicemia [17]. This association was independent of other inflammatory and bone mineral metabolism parameters. In addition, a post hoc analysis of the Cardiovascular Health Study (CHS study) in community-dwelling adults over 65 years (with or without CKD) found that those with high FGF23 levels had a higher rate of first infection-related hospitalization [19]. This association was observed in both patients with CKD and without CKD. These results, including our data, support the potential role of increased baseline FGF23 levels as a risk factor for severe infections.

### 3.3. High FGF23 Levels and Severe Infections: Correlation or Causality?

Previous data have shown that high FGF23 titers are associated with increased rates of cardiovascular events in CKD patients [13,20,21] and the general population [22]. FGF23 has deleterious cardiovascular effects, including cardiac hypertrophy [23,24,25], arterial wall calcification, and alteration of intracellular calcium and smooth muscle cell contractility [26]. There is currently limited evidence concerning the potential mechanisms related to the association of high baseline FGF23 levels and increased risk of severe infections. FGF23 is a negative modulator of the 1-alpha hydroxylase, the enzyme responsible for active vitamin D synthesis (1,25 dihydroxy vitamin D), both in renal [27,28] and extrarenal tissue, including monocytes [29]. Vitamin D is a hormone that modules immune response [30], and low vitamin D levels plus high FGF23 levels (a common condition in ESRD patients) are associated with adverse infectious outcomes [16]. A translational study demonstrated that FGF23 per se causes an immune dysfunction in experimental murine models and white blood cells obtained from healthy volunteers [15,31]. These effects include decreased neutrophil selectin-mediated rolling and chemokine-induced recruitment in vitro. In addition, in experimental models of bacterial pneumonia in CKD mice, the administration of FGF23 exacerbated disease activity and decreased murine survival [15]. Moreover, pharmacological blockage of FGF23 using specific anti-FGF23 antibodies [15] and short-acting small molecules reversibly inhibiting FGF23 [30,32] prevent cardiac and immune effects in vitro and in CKD mice. The mechanisms have not been clarified yet. It has been proposed that FGF23 may activate the FGFR2 receptor in neutrophil cells and cause indirect effects mediated by the FGFR receptors in other cells of the inflammatory milieu [30].

### 3.4. Limitations and Strengths of the Study

Concerning the strengths of this work is the prospective design, which evaluated this group of patients before and during the first two years of the SARS-CoV-2 pandemic, including relevant demographic, clinical, and laboratory baseline data, that was associated with clinical outcomes in ESRD patients. In addition, we had detailed data on COVID-19-related events obtained by direct information from hemodialysis centers and the national COVID-19 database, with follow-up data of all patients during the study period.

In relation to the limitations of this study, its (non-interventional) observational design has biases, as some potential influencing variables are difficult to isolate. For example, people who had a higher incidence of COVID-19, besides higher levels of plasma FGF23, also were older and had increased rates of diabetes, known risk factors for COVID-19 in the general population and ESRD patients [5,33], which may have influenced the differences in clinical outcomes. Additionally, patients with high FGF23 levels also had high PTH levels. PTH is modulated by FGF23 [13], and low PTH levels have been associated with increased infection rates in ESRD patients [34,35]. To decrease this bias, we used statistical adjustments, including potential confounding variables, such as multivariate regressions, to evaluate the association of FGF23 levels and adverse clinical outcomes regardless of other demographical and clinical variables. Another limitation is the lack of data on C-terminal FGF23 levels in these patients. Similar to intact FGF23, high C-terminal FGF23 is associated with worse clinical outcomes and increased mortality [13,14] and has been associated with increased rates of severe infections [17]. Currently, no published data compares intact versus C-terminal FGF23 and its association with severe infections, including COVID-19.

## 4. Conclusions

Our results suggest that high baseline plasma FGF23 levels in end-stage renal disease patients on chronic hemodialysis are associated with an increased risk for SARS-CoV-2 infection and severe COVID-19, including hospitalization and death. This is one of the first reports that evaluate the relationship between FGF23 and COVID-19 in this population. These data support the potential immunosuppressive effects of high circulating FGF23 as a factor associated with worse clinical outcomes in ESRD and non-ESRD patients. Further data from translational research and clinical studies with larger populations are needed to confirm this hypothesis.

## 5. Materials and Methods

### 5.1. Study Design

The CATALINA-HD study is a multicenter observational cohort of end-stage renal disease patients on chronic hemodialysis performed in Chile. The study was initiated in 2018 to evaluate the association of FGF23 with clinical outcomes, including hospitalizations, major cardiovascular events, infectious events, and death. The information included demographic, laboratory, and clinical data, which were collected from patients. Measurements of plasma FGF23 were performed in 2019. We evaluated the association of plasma FGF23 levels before the SARS-CoV-2 pandemic with clinical outcomes, including SARS-CoV-2 infection and severe COVID-19, including COVID-related hospitalizations and deaths. The study was approved by the local Institutional Ethics Committee.

### 5.2. Inclusion and Exclusion Criteria

We included people older than 18 years with a previous diagnosis of end-stage renal disease who were on chronic hemodialysis before 1 January 2020 and had measurements of plasma FGF23 levels during 2019. The analysis included the final measurement if a patient had more than one measurement. We excluded: terminal patients with palliative management, pregnancy or breastfeeding women, and patients with incomplete data.

### 5.3. Evaluation of Patients

Determination of plasma levels of intact FGF23 was performed using an enzyme-linked immunosorbent assay (ELISA) technique (human iFGF23: cat#60–6600, Quidel, San Diego, CA, USA). Blood samples were collected during the hemodialysis session, before the initiation of hemodialysis, by the puncture of the vascular access (arteriovenous fistula or central venous catheter) using a 4-mL EDTA tube. Samples were centrifugated, and plasma was extracted and then used for measurements. Patients were classified according to their plasma FGF23 levels, as low level (FGF23 below p50 of the whole sample) and high level (FGF23 greater than or equal to p50 of the whole sample).

Patient follow-up was carried out between 1 January 2020 and 31 December 2021. SARS-CoV-2 infection was confirmed by a polymerase chain reaction (PCR) test. This information was reported on the Epivigila platform (the method used by the Chilean Ministry of Health to perform patient follow-ups) [36]. We evaluated patients who had COVID-related hospitalization or COVID-related death with PCR confirmation, corresponding to the U07.1 code in the International Classification of Diseases, 10th Revision [37].

### 5.4. Statistical Analysis

Continuous variables are expressed as arithmetic mean ± standard deviation or median [percentile 25—percentile 75], and discrete variables are expressed as absolute values (percentages). To compare baseline data between groups, we used the chi-square test for discrete variables or the Student *t*-test for paired or unpaired groups. In addition, calculations of Hazard Ratios and their 95% confidence intervals (95% CI), Kaplan-Meier analysis, and Cox proportional tests were performed for survival analysis. Finally, we performed a multivariate analysis using logistic regression to evaluate predictors of SARS-CoV-2 infection or severe COVID-19, evaluating several clinical, demographical, and laboratory variables. In those models, we included variables that had a *p*-value below 0.20 in univariate analysis. Data analysis was executed using GraphPad Prism v.6.0 (GraphPad Software, La Jolla, CA, USA) and Stata/SE v.17.0 (Stata Software, College Station, TX, USA). All the analyses were two-tailed, and we considered a statistically significant difference with a *p*-value below 5% (*p* < 0.05).

## Figures and Tables

**Table 1:** Baseline characteristics of patients. Information on the total cohort and stratification by the presence of SARS-CoV-2 infection are presented. Data are expressed as number (N) and percentage (%) for categorical variables and as mean ± standard deviation or median [p25–p75] for continuous variables. The *p*-values of patients who presented SARS-CoV-2 infection vs. those without SARS-CoV-2 infection are detailed.

Characteristics		Total Cohort		Patients without SARS-CoV-2 Infection		Patients with SARS-CoV-2 Infection		*p* Value
		N	%	N	%	N	%	
Total		243	100%	198	81.48%	45	18.52%	-
Sex	Female (%)	120	49.38%	102	51.52%	18	40.00%	0.163
	Male (%)	123	50.62%	96	48.48%	27	60.00%	
Age group	18–39 years (%)	8	3.29%	8	4.04%	0	0.00%	0.024
	40–49 years (%)	37	15.23%	35	17.68%	2	4.44%	
	50–59 years (%)	66	27.16%	56	28.28%	10	22.22%	
	60–69 years (%)	95	39.09%	73	36.87%	22	48.89%	
	70–79 years (%)	31	12.76%	23	11.62%	8	17.78%	
	≥80 years (%)	6	2.47%	3	1.52%	3	6.67%	
Comorbidities	Diabetes (%)	110	45.27%	83	41.92%	27	60.00%	0.028
	Hypertension (%)	218	89.71%	177	89.39%	41	91.11%	0.732
	Heart failure (%)	40	16.46%	31	15.66%	9	20.00%	0.478
Vascular access	Arteriovenous fistula (%)	140	57.61%	111	56.06%	29	64.44%	0.304
	Hemodialysis catheter (%)	103	42.39%	87	43.94%	16	35.56%	
Hemodialysis parameters	Residual diuresis (%)	80	32.92%	67	33.84%	13	28.89%	0.524
	Hemodialysis vintage (months)	25 [15–40]		25 [15–39]		26 [18–45]		0.481
	Dry weight (kg)	70.20 ± 7.66		70.54 ± 7.45		68.71 ± 8.44		0.148
	Single pool Kt/V (spKt/V)	1.31 ± 0.20		1.31 ± 0.21		1.34 ± 0.22		0.413
Medications	Angiotensin receptor blockers (%)	175	72.02%	142	71.72%	33	73.33%	0.827
	Calcium channel blockers (%)	178	73.25%	147	74.24%	31	68.89%	0.464
	Loop diuretics (%)	46	18.93%	35	17.68%	11	24.44%	0.296
	Vitamin D analogs (%)	56	23.05%	46	23.23%	10	22.22%	0.885
	Phosphate binders (%)	209	86.01%	170	85.86%	39	86.67%	0.888
	Calcimimetics (%)	48	19.75%	40	20.20%	8	17.78%	0.712
	Erythropoietic stimulating agents (%)	203	83.54%	166	83.84%	37	82.22%	0.792
Laboratory parameters	Blood ureic nitrogen (mg/dL)	64.06 ± 12.70		64.56 ± 12.31		61.86 ± 14.25		0.199
	Intact parathormone (pg/mL)	565 [284–884]		572 [275–888]		511 [293–795]		0.433
	25-OH vitamin D (ng/mL)	19.05 ± 8.53		19.31 ± 8.57		17.93 ± 8.36		0.328
	Serum phosphate (mg/dL)	5.15 ± 1.08		5.16 ± 1.12		5.08 ± 8.78		0.664
	Total serum calcium (mg/dL)	8.22 ± 0.98		8.21 ± 0.98		8.26 ± 1.01		0.785
	Ferritin (ng/mL)	467.72 ± 168.45		464.82 ± 170.17		480.51 ± 161.89		0.573
	Hemoglobin (g/dL)	9.45 ± 1.33		9.42 ± 1.36		9.61 ± 1.20		0.379
	Intact fibroblast growth factor 23 (pg/mL)	319 [204–600]		288 [195–580]		436 [269–669]		0.026

**Table 2:** Comparison of patients according to plasma FGF23 levels. Information on the total cohort and stratification by baseline plasma intact FGF23 levels are presented. Data are expressed as number (N) and percentage (%) for categorical variables and as mean ± standard deviation or median [p25–p75] for continuous variables. *p*-values of low FGF23 vs. high FGF23 are detailed.

Characteristics		Total Cohort		Patients with Low FGF23 Levels (<p50)		Patients with High FGF23 Levels (≥p50)		*p*-Value
		N	%	N	%	N	%	
Total		243	100%	121	49.79%	122	50.21%	-
Sex	Female (%)	120	49.38%	57	47.11%	63	51.64%	0.480
	Male (%)	123	50.62%	64	52.89%	59	48.36%	
Age group	18–39 years (%)	8	3.29%	6	4.96%	2	1.64%	0.236
	40–49 years (%)	37	15.23%	22	18.18%	15	12.30%	
	50–59 years (%)	66	27.16%	29	23.97%	37	30.33%	
	60–69 years (%)	95	39.09%	50	41.32%	45	36.89%	
	70–79 years (%)	31	12.76%	12	9.92%	19	15.57%	
	≥80 years (%)	6	2.47%	2	1.65%	4	3.28%	
Comorbidities	Diabetes (%)	110	45.27%	49	40.50%	61	50.00%	0.137
	Hypertension (%)	218	89.71%	109	90.08%	109	89.34%	0.850
	Heart failure (%)	40	16.46%	19	15.70%	21	17.21%	0.751
Vascular access	Arteriovenous fistula (%)	140	57.61%	74	61.16%	66	54.10%	0.266
	Hemodialysis catheter (%)	103	42.39%	47	38.84%	56	45.90%	
Hemodialysis parameters	Residual diuresis (%)	80	32.92%	42	34.71%	38	31.15%	0.555
	Hemodialysis vintage (months)	25 [15–40]		27 [16–42]		23 [14–39]		0.128
	Dry weight (kg)	70.20 ± 7.66		70.47 ± 7.74		69.92 ± 7.60		0.574
	Single pool Kt/V (spKt/V)	1.31 ± 0.20		1.35 ± 0.21		1.27 ± 0.20		0.003
Medications	Angiotensin receptor blockers (%)	175	72.02%	92	76.03%	83	68.03%	0.165
	Calcium channel blockers (%)	178	73.25%	91	75.21%	87	71.31%	0.493
	Loop diuretics (%)	46	18.93%	23	19.01%	23	18.85%	0.975
	Vitamin D analogs (%)	56	23.05%	29	23.97%	27	22.13%	0.734
	Phosphate binders (%)	209	86.01%	107	88.43%	102	83.61%	0.279
	Calcimimetics (%)	48	19.75%	23	19.01%	25	20.49%	0.771
	Erythropoietic stimulating agents (%)	203	83.54%	95	78.51%	108	88.52%	0.035
Laboratory parameters	Blood ureic nitrogen (mg/dL)	64.06 ± 12.70		62.86 ± 13.56		65.24 ± 11.73		0.145
	Intact parathormone (pg/mL)	565 [284–884]		379 [233–710]		697 [479–918]		0.001
	25-OH vitamin D (ng/mL)	19.05 ± 8.53		19.14 ± 8.12		18.96 ± 8.96		0.868
	Serum phosphate (mg/dL)	5.15 ± 1.08		5.17 ± 1.13		5.12 ± 1.03		0.725
	Total serum calcium (mg/dL)	8.22 ± 0.98		8.24 ± 1.05		8.19 ± 0.92		0.668
	Ferritin (ng/mL)	467.72 ± 168.45		472.89 ± 167.94		462.60 ± 169.49		0.635
	Hemoglobin (g/dL)	9.45 ± 1.33		9.36 ± 1.35		9.55 ± 1.31		0.263
	Intact fibroblast growth factor 23 (pg/mL)	319 [204–600]		204 [160–267]		598 [465–703]		<0.001
Clinical outcomes	SARS-CoV-2 infection (%)	45	18.52%	16	13.22%	29	23.77%	0.034
	COVID-19-related hospitalization (%)	35	14.40%	12	9.92%	23	18.85%	0.047
	COVID-19-related death (%)	12	4.94%	4	3.31%	8	6.56%	0.242
	COVID-19-non-related death (%)	20	8.23%	8	6.61%	12	9.84%	0.360
	COVID-19-related hospitalization or death (%)	36	14.81%	12	9.92%	24	19.67%	0.032

**Table 3:** Determination of predictors of SARS-CoV-2 infection during a 2-year follow-up. We present a univariate and multivariate logistic regression of several clinical, demographic, and laboratory parameters of patients. The variables which were included in the multivariate model were those which had a *p*-value below 0.2 in the univariate analysis. Variables that were considered predictors of SARS-CoV-2 infection were those with a *p*-value below 0.05 in the final model. Hazard ratios, the 95% confidence interval (95%CI), and the *p*-value are detailed.

	Univariate Analysis				Multivariate Analysis			
Variable	Hazard Ratio	95% CI		*p*-Value	Hazard Ratio	95% CI		*p*-Value
Male	1.551	0.854	2.816	0.149	1.597	0.875	2.915	0.127
Age > 60 years	2.634	1.360	5.101	0.004	2.630	1.352	5.118	0.004
Diabetes	1.909	1.051	3.465	0.034	1.916	1.053	3.485	0.033
Hypertension	1.192	0.427	3.328	0.737				
Heart failure	1.315	0.634	2.731	0.462				
Vascular access (fistula)	1.415	0.768	2.604	0.265				
Residual diuresis	0.819	0.430	1.561	0.545				
Hemodialysis vintage	1.004	0.991	1.017	0.568				
Dry weight	0.973	0.938	1.008	0.128	0.981	0.946	1.017	0.301
spKt/V	1.904	0.469	7.738	0.368				
Use of ARBs	1.076	0.556	2.083	0.829				
Use of CCBs	0.798	0.424	1.500	0.483				
Use of loop diuretics	1.387	0.702	2.737	0.346				
Use of vitamin D analogs	0.959	0.475	1.938	0.908				
Use of phosphate binders	1.073	0.454	2.534	0.873				
Use of calcimimetics	0.899	0.419	1.931	0.786				
Use of ESAs	0.840	0.391	1.803	0.654				
Blood ureic nitrogen	0.987	0.963	1.010	0.261				
Intact PTH	1.000	0.999	1.000	0.420				
25-OH vitamin D	0.982	0.948	1.016	0.295				
Serum phosphate	0.941	0.722	1.226	0.654				
Total serum calcium	1.060	0.789	1.425	0.697				
Ferritin	1.000	0.999	1.002	0.580				
Hemoglobin	1.091	0.884	1.347	0.418				
Intact FGF23 (>p50)	1.917	1.041	3.530	0.037	1.920	1.035	3.562	0.039

**Table 4:** Determination of predictors of severe COVID-19 during a 2-year follow-up. We present a univariate and multivariate logistic regression of several clinical, demographic, and laboratory variables of patients. Severe COVID-19 is defined as the requirements for hospitalizations or COVID-related deaths. The variables which were included in the multivariate model were those which had a *p*-value below 0.2 in the univariate analysis. Variables that were considered predictors of severe COVID-19 were those which had a *p*-value below 0.05 in the final model. Hazard ratios, the 95% confidence interval (95%CI), and the *p*-value are detailed.

	Univariate Analysis				Multivariate Analysis			
Variable	Hazard Ratio	95% CI		*p*-Value	Hazard Ratio	95% CI		*p*-Value
Male	1.823	0.923	3.600	0.084	1.913	0.963	3.800	0.064
Age > 60 years	2.841	1.336	6.043	0.007	2.655	1.221	5.776	0.014
Diabetes	2.546	1.273	5.091	0.008	2.526	1.259	5.066	0.009
Hypertension	0.913	0.323	2.583	0.865				
Heart failure	1.749	0.822	3.719	0.147	1.433	0.660	3.110	0.363
Vascular access (fistula)	1.223	0.626	2.390	0.556				
Residual diuresis	0.668	0.314	1.420	0.294				
Hemodialysis vintage	1.005	0.991	1.019	0.496				
Dry weight	0.970	0.932	1.010	0.139	0.979	0.940	1.020	0.319
spKt/V	2.258	0.472	10.809	0.308				
Use of ARBs	0.879	0.432	1.786	0.721				
Use of CCBs	0.718	0.359	1.435	0.348				
Use of loop diuretics	1.242	0.566	2.725	0.589				
Use of vitamin D analogs	1.111	0.523	2.363	0.784				
Use of phosphate binders	1.017	0.395	2.615	0.972				
Use of calcimimetics	1.021	0.447	2.330	0.961				
Use of ESAs	0.755	0.331	1.724	0.505				
Blood ureic nitrogen	0.984	0.959	1.011	0.239				
Intact PTH	1.000	0.999	1.001	0.866				
25-OH vitamin D	0.982	0.945	1.021	0.363				
Serum phosphate	0.869	0.645	1.171	0.356				
Total serum calcium	1.125	0.807	1.568	0.487				
Ferritin	1.001	0.999	1.003	0.558				
Hemoglobin	1.034	0.813	1.315	0.783				
Intact FGF23 (>p50)	2.116	1.058	4.232	0.034	2.121	1.049	4.287	0.036

## Data Availability

The information that supports the findings of our study is available upon reasonable request to the corresponding author, L.T. This information is not publicly available because it contains data that may compromise the privacy of the research participants and third-party restrictions.
